# The Germinal Center Milieu in Rheumatoid Arthritis: The Immunological Drummer or Dancer?

**DOI:** 10.3390/ijms221910514

**Published:** 2021-09-29

**Authors:** Dornatien C. Anang, Giulia Balzaretti, Antoine van Kampen, Niek de Vries, Paul L. Klarenbeek

**Affiliations:** 1 Department of Rheumatology & Clinical Immunology, Amsterdam Rheumatology & Immunology Center, 1007 MB Amsterdam, The Netherlands; d.c.anang@amsterdamumc.nl (D.C.A.); g.balzaretti@amsterdamumc.nl (G.B.); p.l.klarenbeek@amsterdamumc.nl (P.L.K.); 2 Department of Experimental Immunology, Amsterdam Infection & Immunity Institute, Amsterdam UMC, University of Amsterdam, 1105 AZ Amsterdam, The Netherlands; 3 Department of Epidemiology and Data Science, Amsterdam Public Health Research Institute, Amsterdam Infection & Immunity Institute, Amsterdam UMC, University of Amsterdam, 1105 AZ Amsterdam, The Netherlands; a.h.vankampen@amsterdamumc.nl; 4 Department of Rheumatology, Spaarne Gasthuis, Hoofdorp, 2000 AK Haarlem, The Netherlands

**Keywords:** Rheumatoid Arthritis, autoantibodies, lymphoid organs, germinal centers, follicular T cells

## Abstract

Rheumatoid Arthritis (RA) is a chronic autoimmune disease characterized by joint inflammation, affecting approximately 1% of the general population. To alleviate symptoms and ameliorate joint damage, chronic use of immunosuppressives is needed. However, these treatments are only partially effective and may lead to unwanted side effects. Therefore, a more profound understanding of the pathophysiology might lead to more effective therapies, or better still, a cure. The presence of autoantibodies in RA indicates that B cells might have a pivotal role in the disease. This concept is further supported by the fact that a diverse antibody response to various arthritis-related epitopes is associated with arthritis development. In this context, attention has focused in recent years on the role of Germinal Centers (GCs) in RA. Since GCs act as the main anatomic location of somatic hypermutations, and, thus, contributing to the diversity and specificity of (auto) antibodies, it has been speculated that defects in germinal center reactions might be crucial in the initiation and maintenance of auto-immune events. In this paper, we discuss current evidence that various processes within GCs can result in the aberrant production of B cells that possess autoreactive properties and might result in the production of RA related autoantibodies. Secondly, we discuss various (pre-)clinical studies that have targeted various GC processes as novel therapies for RA treatment.

## 1. Introduction

RA is a common autoimmune disease that affects about 1% of the worlds’ population [1,2]. Despite the fact that the disease pathogenesis is poorly understood, the clinical efficacy of B cell-directed therapy (BCDT) as well as findings that B cell depletion can delay onset of the disease underscore the concept that B cells are likely to play a pivotal role in the disease process [3,4]. Additionally, the finding that epitope spreading of the antibody response is associated with disease onset and disease course in early arthritis suggest that a more diverse antibody and B cell immune response is crucial for disease development [5,6]. This hypothesis suggests that the key to understanding the role of B cells in RA lies at an earlier stage of B cell development and differentiation. The question of when and where these B cells of varied specificities arise has largely remained unanswered for decades. Interestingly, somatic hyper mutation (SHM) of V(D)J genes, which is known to be the major contributor to Immunoglobulin (Ig) diversity and specificity, occurs primarily within GCs [7]. Whether the process of SHM and other processes within GCs are the main drivers of autoimmunity is yet to be fully explored. Therefore, there is an urgent need to unravel in detail the GC responses, as this might be the origin of not only anti-citrullinated protein antibodies (ACPA) producing B cells but also other B cells of unknown specificities that largely contribute to the chronic inflammatory process in RA as well as other autoimmune diseases. 

GCs have been described as specialized structures formed within secondary lymphoid organs (SLOs), such as lymph nodes (LNs) and spleen, where B cell clonal expansion and affinity maturation takes place to increase B cell diversity and affinity for foreign antigen [8,9,10,11,12]. In the course of these processes, inevitable by-products are “undesirable” autoreactive B cell clones to self-antigens that might elicit an autoimmune response [13,14]. These B cells have been termed “rogue” B cells [15,16], and although it is widely speculated that these rogue B cells arise from defects in central and peripheral tolerance, several questions have largely remained unanswered to immunologists, including (1) the precise anatomic origin of these rogue B cells, (2) the exact role of autoantigens in eliciting the autoimmune responses and (3) why different patients have different autoantibodies. This has made the selective therapeutic targeting of these “rogue” B cells more challenging, if not impossible at the moment. More recently, compelling evidence has pointed to GCs as a possible source of these autoreactive B cells in RA and other autoimmune disorders [17,18,19,20,21]. More so, the findings that reverting most autoantibodies to their germline configuration results in loss of their reactivity towards their target antigens as well as recent advances in high throughput technologies that permit a high-resolution dissection of GCs and its components has spurred enormous interest into GC biology as a potential culprit in the generation of these autoantibodies [22,23]. Therefore, a deeper understanding of GC responses in the context of autoimmunity is needed and could be a next step towards modulating those immune changes that are a major signature of these diseases. 

In this review, we briefly describe the physiological role of GCs in adaptive immune responses. Thereafter, we discuss emerging evidence that implicates GCs in the pathogenesis of RA. Next, we look at various cellular subsets resident within GCs and how they could contribute in driving the autoimmune response. Finally, we end by looking at novel approaches targeting GC processes as therapeutic possibilities in the treatment of RA. 

## 2. Germinal Centers and Adaptive Immune Responses

**Figure 1:**
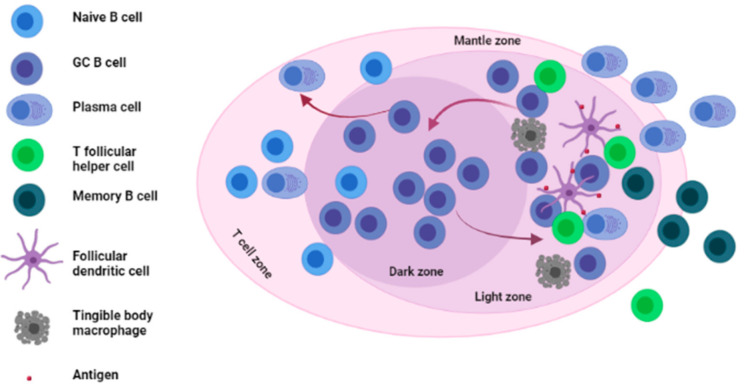
Architecture and main cellular components of the germinal center. The germinal center is mainly divided into two zones; the Dark zone (DZ) where GC B cells undergo somatic hypermutation (SHM) of their immunoglobulin (Ig) genes and the light zone (LZ) where GC B cells interact with antigens presented to them by FDCs as well as T follicular helper cells (Tfh). GC: germinal center, FDCs: follicular dendritic cells.

Before we evaluate evidence on the role of GCs in RA, we will briefly describe the physiological role and importance of GCs in adaptive immune responses. GCs are specialized structures formed within LNs, spleen and other SLOs where affinity selection, clonal expansion and SHM of B cells takes place [24,25,26] (see Figure 1). The formation and role of GCs, which has been reviewed extensively elsewhere, is known to occur in B cell-rich follicles via an antigen-driven mechanism [17,27,28]. Briefly, in the event of a normal immune response against foreign antigens, naïve B cells, upon encountering antigens, migrate to the T cell–B cell border of lymphoid organs. Thereafter, B cells, which continue to respond to antigen signals, move to the center of the lymphoid follicles to form what is known as the nascent GC [29]. Following the development of this nascent GC into a matured GC, two major anatomical features of GCs (dark zone (DZ) and light zone (LZ)) are formed [10,30]. These zones are highly crucial for GC reactions. In the DZ, B cells undergo extensive B cell clonal expansion and SHM of the Ig genes to increase B cell diversity and to change affinity. Upon exit to the LZ, B cells undergo positive and negative selection based on their affinity for antigen presented by follicular dendritic cells (FDCs) as well as their interaction with T follicular helper (Tfh) cells [31,32]. Positively selected B cells can either be recycled back to the DZ, where they undergo further rounds of clonal expansion and SHM, or undergo differentiation into memory B cells or plasma cells, which subsequently exit the GC via the LZ or DZ (in the case of plasma cells) into the periphery [17,33,34]. The recycling of positively selected B cells from the LZ back into the DZ termed “cyclic re-entry” enables efficient affinity maturation and generation of B cell clones with very high affinity for foreign antigen [35]. On the other side, low affinity or autoreactive B cells in the LZ are deleted via apoptosis mediated in part by tingible body macrophages (TBMs) located in the LZ [17]. Plasma cells and memory B cells that result from GC reactions are often long-lived and migrate to peripheral structures, such as bone marrow in the case of plasma cells, where they access survival niches that permits them to live for months if not years [36].

Unfortunately, the checkpoint mechanisms within GCs are not perfect, resulting in the occasional selection of auto-reactive B-cell clones that escape into the periphery [37]. Because many autoimmune diseases are characterized by autoreactive antibodies it can be hypothesized that problems in the GC checkpoint might lead to autoimmune disease [27].

## 3. Germinal Centers in Rheumatoid Arthritis

There are several lines of evidence that implicate (ectopic) GCs in the process of autoantibody generation in RA. First and foremost are studies that have directly identified GCs in various tissues such as synovial tissues and lymph nodes of RA patients (discussed hereafter). A second line of evidence comes from examining RA-related autoantibodies. Multiple studies have demonstrated the presence of extensive SHM in these autoantibodies, which is a hallmark of a GC reaction. Thirdly, several groups have described defects in GC processes that result in features associated with auto-immunity. We will discuss these three findings in detail below.

### 3.1. The Presence of GCs and Ectopic GCs in Tissues of RA Patients and Mouse Models of Arthritis

#### 3.1.1. The Presence of GCs and Ectopic GCs in Tissues of RA Patients

While traditional GCs have gained enormous interest in recent years, ectopic GCs have been reported in about 40–60% of RA patients as well [38,39]. These ectopic GCs, which form primarily at sites of inflammation, are known to differ from traditional GCs in their vasculature, microenvironment and composition. In the literature, these ectopic GCs are thought to intensify or continue the autoimmune response outside the follicular region or arise as a feedback mechanism aimed at censoring the autoreactive B cell clones that arise as by-products from traditional GC reactions [40,41]. Both GCs and ectopic GCs have been implicated in the pathogenesis of RA. The findings that rheumatoid factors (RFs) could be found within GCs in lymph nodes obtained from RA patients more than fifty years ago was one of the first pieces of evidence of a possible involvement of GCs in the development of RA [42]. Another indication of GC involvement in RA was reported in the late 1980s. Using microscopy and immunohistochemistry, Imai et al. reported increased numbers of GCs in lymph nodes and synovial tissues of RA patients [43]. In recent years, evidence has come from observations in synovial tissues and lymph nodes of RA patients [18,44]. In one of such studies, microdissection and sequencing of V_H_/V_L_ chains of synovial tissue ectopic GC-derived B cells revealed a high degree of clonally related plasma cells, suggesting the possibility that these cells underwent terminal differentiation in the inflamed synovium of this patients. These results indicate that local GCs as well as ectopic GCs may, indeed, contribute to the pool of antibody-producing plasma cells that accumulate at a local site of inflammation.

#### 3.1.2. The Presence of GCs and Increased GC Responses in Tissues of RA Mouse Models

In addition to observations in human RA, findings from animal models of arthritis have reported clues on the role of GCs in animal models of RA. In the well-characterized collagen-induced arthritis (CIA) mouse model of arthritis, the development of autoimmune arthritis was highly associated with an increased GC response in lymph node tissues [45]. Interestingly, an increased GC response in these mice correlated with an increased number of GC-B cells as well as an increased production of anti-collagen antibodies. In another study involving an antigen-induced arthritis (AIA) mouse model, a K/BxN serum-transfer–induced arthritis (STIA) mouse model, and CIA mice, an increased traditional GC response was observed in all mouse models compared to wild type controls and, inhibiting GC reactions resulted in a marked decrease in antibody-driven arthritis [46]. Finally, several other studies from Han et al., Hou et al., and Wang et al. have all reported similar results implicating GCs and GC reactions in the disease process in animal models of RA [47,48,49].

### 3.2. SHM within GCs and the Generation of RA-Related Autoantibodies

Another finding that implicates GCs in RA development is the presence of SHM in RA-related autoantibodies. SHM, a process known to occur on centroblasts within GCs often results in the accumulation of point mutations in the Ig variable genes of antibodies. While SHM often results in the production of B cell clones with high affinity B cell receptors (BcRs), the process of SHM is not able to distinguish between “favorable” and “unfavorable” mutations. It is the resulting BcRs affinity that is the basis for affinity selection. Therefore, B cells with varied BcR affinities and specificities result from this process.

As the SHM process is stochastic in nature, a possible outcome could be the ‘accidental’ generation and positive selection of B cell clones with high affinity for self-antigens [50,51]. The subsequent exit of such high affinity self-reactive B cell clones from the GC into the periphery could result in the initiation of autoimmune events. A compelling example outlining this possibility was a study by Guo et al., which reported the creation of high-avidity autoimmune B cells from non-autoimmune precursors by the process of SHM targeted in V region genes of anti-nuclear antibody (ANA) producing B cells [50]. In the context of RA, a strong correlation between SHM and the generation of ACPAs came from a report by Li et al., where the presence of highly somatically mutated Ig genes encoding ACPAs in RA patients was reported. More interesting, reverting the variable regions of these ACPA encoding Ig genes to their respective germline genes resulted in loss of their reactivity towards citrullinated RA antigens further indicating a possible GC origin of these antibodies [52].

While the studies discussed above directly implicate the process of SHM in autoantibody generation, there are additional findings on Activation-induced Cytidine Deaminase (AID), which is a key enzyme in regulating SHM that implicates SHM in autoimmunity. In a group of RA patients, an increased expression of AID was reported in synovial tissues and peripheral blood [53]. Additionally, a correlation was observed between AID expression and the production of IgG and IgM RF and other autoantibodies against citrullinated peptides. Two other studies have also described the presence of cells which carrySHMs in fibroblast-like synoviocytes (FLS) and synovium of RA individuals [32,54]. Additionally in a study involving BXD2 mice, a mouse model for autoimmune lupus, AID was shown to be highly expressed in splenic tissues and the upregulation could be correlated with the generation of pathogenic autoantibodies in GCs in these mice [49].

Finally, it is important to note that while the process of SHM can largely contribute to the generation of autoreactive B cells, recent evidence indicates that point mutations introduced by SHM on the other hand can circumvent autoantibody production by redeeming self-reactive B cells recruited into GCs [55,56]. Further investigation is needed to explore this possibility.

### 3.3. Defective GC-Resident Cellular Subsets and the Generation of Autoantibodies in RA

Although GCs are difficult to study as they are multicellular structures, there is evidence that defects in GCs are associated with auto-immunity. Here, we will discuss the available observations on Tfh and Tfr cells as these GCs cells have received much interest recently in the context of RA and seem likely candidates for targeted therapeutic strategies.

#### 3.3.1. Defective Tfh Cell Functioning within GCs

Within GCs, Tfh cells are known to be crucial players in the process of affinity selection, which takes place in the LZ. This function is mediated through ICOS (a CD28 family receptor expressed on their surface), CD40L and through their signature cytokines IL-21 and IL-4 [57]. Limiting the number of Tfh cells within a GC reaction was observed to be crucial in the selection of B cells with high affinity BcRs [58]. Therefore, it seems that Tfh quantity and quality are a major limiting factor for B cell selection within GCs. Hence, aberrant or excessive Tfh signals within GCs could result in a GC environment characterized by reduced competition from B cells. The outcome is the selection of B cells carrying both “normal” as well as “autoreactive” BcRs.

There is evidence of such aberrant Tfh numbers in various RA mouse models. Recently, Cao and colleagues reported an increased frequency of Tfh cells in CIA mice compared to unimmunized animals. Furthermore, this increase was associated with elevated levels of (interleukin-21) IL-21, a signature cytokine produced by Tfh cells [59]. Independently, two groups demonstrated the role of Tfh cell-associated markers (CXCR5 and adaptor SLAM-associated protein (SAP)) in the induction and maintenance of arthritis in CIA as well as SAP^−/−^ KRN-Tg mice models. In both studies, arthritis was prevented when these molecules were targeted [60,61]. In addition, another study in K/BxN mouse described the importance of IL-21, a typical Tfh cytokine in the development of arthritis. In this study, an induced deficiency in the receptor of IL-21 was sufficient to completely prevent the onset and subsequent development of arthritis in the K/BxN mouse model. This clearly attributes an important role of the IL-21/IL-21R axis in the very early stages of arthritis development [62].

It is worth mentioning that unlike in mouse models, difficulties do exist in assessing GC-Tfh cells in humans due to their relatively low numbers coupled with obstacles in tissue sampling. Therefore, the circulatory form of these cells termed “Circulatory T follicular helper cells (cTfh)” have been extensively described as a counterpart of Tfh cells in GCs [63,64]. Data from human studies on cTfh cells in RA patients clearly corroborate mouse data on aberrant Tfh numbers (Table 1). Several studies have reported an increase in cTfh cells in RA patients, accompanied by elevated IL-21 levels in some cases [65,66,67,68,69,70,71]. However, it is important to note that while there are increase Tfh numbers both in murine and human GCs, the underlying mechanism responsible for their expansion remains to be fully unraveled. Could antigen quantity and quality within GCs be responsible for their expansion? Or do these cells expand already in the periphery prior to their migration into GCs? These are some of the questions that will need to be answered.

#### 3.3.2. Defective Tfr Cell Functioning within GCs

In recent years, a regulatory GC-resident T cell subset termed T follicular regulatory cells (Tfr) has been in the spotlight. Similar to Tfh cells, Tfr cells express CXCR5, Bcl-6, ICOS and PD-1 coupled with their expression of Foxp3 and CTLA-4, which enables them to be distinguished from Tfh cells [75,76]. Since their initial discovery more than a decade ago, a major point of contention has been the development and maturation process of Tfr cells. While an earlier study from Linterman and colleagues suggested that thymic Tregs have the ability to convert into Tfr cells [77], this was disputed by two other studies which reported the absence of Tfr cells in the thymus [78,79]. 

Tfr cells have generally been attributed with regulating immune reactions in the GC by dampening excessive GC responses through their interactions with Tfh cells [80]. On the other side, there is evidence of their ability to enhance GC reactions by helping B cells through the release of IL-10 [81]. The mechanism by which Tfr cells can execute both functions of suppressing autoimmunity and promoting B cell help simultaneously have largely remained unexplored. Nevertheless, accumulated evidence has demonstrated that abnormal Tfr cells may result in an imbalance immune milieu that favors the development of autoimmune diseases [79,82,83,84,85]. Two studies exploring the contribution of Tfr cells in RA reported similar findings on elevated Tfr levels in RA patients compared to healthy controls [72,73]. In addition, the regulatory function of Tfr cells was revealed in a recent study by Liu et al., who found an association of increased Tfr cells with decreased autoantibody production in RA patients in stable remission [74]. 

Finally, while their name suggests a cell type involved in immune regulation, it is not clear as to whether the reported elevated levels of Tfr in RA patients is a feedback mechanism aimed at dampening potential “abnormal” Tfh responses in the GCs or on their own contribute to the disease pathogenesis. The rationale of the last option is supported by the observation that a regulatory cell type on its own could be potential pathogenic in another milieu, as has been reported in giant cell arteritis [86].

## 4. Interfering with GC Components as Targets in the Treatment of RA

Considering both direct and circumstantial evidence discussed above on the role of GCs in RA development, recent years have seen an increase in research into potential therapeutic candidates targeting various GC components. However, since plasma cells which are the major producers of autoantibodies do not require GCs for their survival, it is logical to admit that such therapies targeting plasma cell production sites could be suitable during the very early phases such as the pre-clinical phase of RA to prevent the generation of “autoreactive plasma cells” or used to prevent disease relapses in patients.

### 4.1. Interference with Various GC Processes

In pre-clinical studies, treatment of RA via interfering with various processes within GCs looks promising. For instance, in the well characterized CIA mouse model, use of a TANK-binding kinase (a kinase highly involved in the maturation of GC Tfh cells) inhibitor was sufficient in abolishing antibody-mediated CIA by decreasing the production of GC-related autoantibodies [46], while in another study, artemisinin, an antimalarial drug, prevented the development of arthritis in K/BxN mice by inhibiting the formation of autoantibodies that were thought to originate from GCs in this mice [48]. Another interesting outcome from this study was the selective inhibition of GC B cells by artemisinin. These findings suggest a possible selective targeting of proliferating GC B cells as a therapeutic option in the treatment of RA. This would be an attractive therapy, if proven in clinical studies, as this might circumvent the need for total B cell depletion as well as a complete GC interference. However, it is important to keep in mind that the complete shutdown of GC reactions on its own could be deleterious. Therefore, laboratory studies will need to confirm if the benefits outweigh the side effects of these kinds of therapies.

Beside the studies described above, several candidate GC-cellular subsets have been considered as suitable therapeutic targets to dampen abnormal GC reactions in the context of autoimmunity. Although recent interest has mainly been directed towards GC-resident Tfh cells, Tfr cells also offer a window which should be exploited as well. Their ability to regulate GC reactions makes them a potential therapeutic candidate in re-enforcing or regulating abnormal GC reactions. Therefore, we speculate that strategies aimed at increasing Tfr cell frequencies in GCs could be beneficial in regulating the GCs as a decreased frequency of Tfr has been reported in most rheumatic and autoimmune diseases.

### 4.2. Targeting Tfh Cells for the Treatment of RA

**Figure 2:**
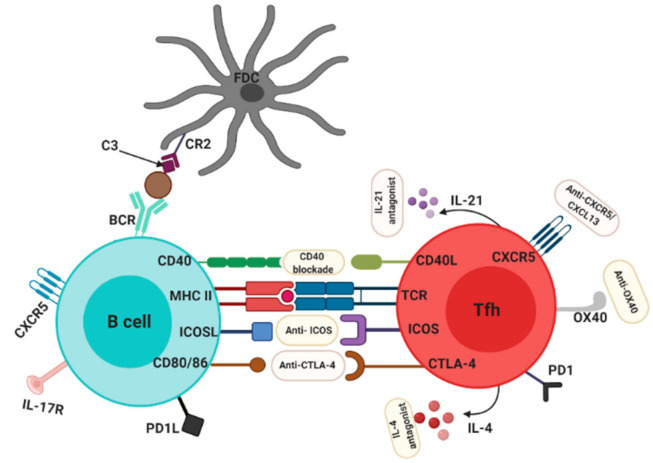
Possible therapeutic targets on Tfh cells for the treatment of RA. Several molecules and receptors expressed on Tfh can be targeted to dampen aberrant immune responses in RA such as anti-ICOS, anti-CTLA4, anti-OX40, anti-CXCR5, Anti-CD40, IL-21 antagonist and IL-4 antagonist. CR2, complement receptor 2; FDC, follicular dendritic cell.

Various approaches could be employed to target Tfh cells as shown in Figure 2. For example, in the treatment of type I diabetes patients with rituximab B cell depletion therapy (chimeric IgG1 anti-CD20), a reported depletion of Tfh cells was associated with a decrease in autoantibody production and decrease in disease severity in these patients [87]. Although Tfh cells are not the primary targets of rituximab, it remains to be determined how rituximab resulted in the depletion of this cells. In other autoantibody-mediated diseases, treatment with abatacept (a CTLA4-Fc IgG1 fusion protein) resulted in the depletion of Tfh cells, which correlated with an improved disease activity [88,89]. In multiple sclerosis patients treated with abatacept the frequency of Tfh cells was also selectively decreased [90]. With evidence from other autoimmune diseases coupled with the role of Tfh cells in GC reactions, it is but logical that strategies that targetTfh cells could be beneficial in RA. This has led to renewed interest in human and murine models and thus far, results look promising.

In different pre-clinical RA mouse models, specific deletion of GC-Tfh cells was shown to be beneficial as the onset of autoimmune arthritis was prevented in these mice [91]. In another study, a more selective approach was employed by T cell-specific deletion of CXCR5. In this study, deletion of CXCR5 induced resistance to arthritis development [61]. This approach, in particular, looks robust and promising in that CXCR5 is expressed solely by Tfh cells, Tfr cells and some B cells. Hence, specific targeting of this molecule leaves a large part of the adaptive immune system relatively unaffected. In a similar approach, Vaccinex, a New York-based biotech company, recently generated a monoclonal antibody (Mab 5261) which specifically targets CXCL13, the main chemokine for CXCR5 and reported a high efficacy in CIA and EAE mouse models [92]. Although Tfh cells were shown to be depleted in humans on the administration of conventional RA therapies [70,90], so far and to the best of our knowledge, no study has attempted a new therapeutic compound in humans which is aimed at specific Tfh depletion. This in fact opens a new therapeutic possibility which remains to be explored in humans.

### 4.3. Tfh-Associated Cytokines as Targets in the Treatment of RA: New Biologics?

The enormous investigations on the therapeutic potential of Tfh cells has resulted in the evaluation of Tfh-associated cytokines as targets in the treatment of RA. IL-21 and IL-4 are the most dominant cytokines produced by Tfh cells for B cell help and seem to be a promising target in inhibiting Tfh activity. IL-21 was observed to be higher in inflamed tissues of RA patients compared to controls, and in various RA mouse models similar observations were reported [93]. Since IL-21 seems to be a signature cytokine of Tfh in GCs, there is a need to unravel its therapeutic potential in the prevention and treatment of autoimmune diseases. A study by Young et al. described decreased levels of IgG1 autoantibodies in DBA/1 mice by blocking the IL-21/IL-21R pathway [94]. In K/XBN mice, use of an IL-21R antagonist abrogated arthritis onset [62], and in mouse CIA, a similar beneficial effect was observed when the IL-21R was blocked during the induction phase of arthritis [95]. Besides mouse reports, several in vitro studies have reported a beneficial effect of IL-21 blockade. For example, in synovial membrane cells in culture, the addition of an IL-21R-FC blocking agent resulted in a decreased production of pro-inflammatory cytokines [96]. In another study where IL-21 was associated with osteoclastogenesis in synovial tissues of RA patients, IL-21 blockade was proposed as a possible therapeutic approach to prevent osteoclastogenesis [97].

### 4.4. Targeting Tfh Activation and Co-Stimulation Pathways in the Treatment of RA

Another aspect of the biology of Tfh cells that has been explored therapeutically in RA involves the activation and co-stimulatory pathway [98]. In this regards, the CD40/CD40L pathway, the OX40/OX40L pathway and, the ICOS/ICOSL pathway have been the focus of numerous studies [99]. The CD40 Ligand (CD40L) is a member of the Tumor necrosis factor (TNF) superfamily whose expression on T cells is known to be crucial for Tfh cells to carry out their helper functions within GCs. Engagement of CD40L to its receptor CD40 on B cells leads to isotype class switching, proliferation and formation of plasma cells and memory B cells [100]. Evidence of CD40L involvement in RA has been presented in several studies. For instance, T cells from RA patients were shown to express high levels of CD40L [101,102] and, another study demonstrated the necessity of CD40 signaling for the production of RF [103]. In addition, a study in CIA mice demonstrated the vital role CD40 plays in the initiation of arthritis [104] and in another RA mouse model, targeting CD40 was enough to prevent the onset of arthritis [105]. Taken together, these studies and many more provide a basis for targeting the CD40L/CD40 pathway in the treatment of RA. In a recently published phase IIa double-blind clinical trial (NCT01751776) with 67 RA patients treated with BI 655064, a CD40/CD40L pathway inhibitor caused marked changes in various biological and clinical parameters including reduced autoantibody production and inflammation, reduced activated B cells as well as a reduction in bone resorption although a favorable clinical efficacy could not be observed [106]. Besides reduced autoantibody levels, a relatively positive outcome from this study was the safety profile that was observed unlike previous trials involving CD40 blockade that were terminated prematurely due to adverse side effects. In accordance with the previous study, a phase Ib proof-of-concept trial involving another CD40 antagonist (VIB4920) in RA patients reported beneficial results with a marked decrease in disease activity [107]. Although the results obtained so far look promising, further research is needed to fully evaluate the clinical safety and efficacy of CD40L blockade in the treatment of RA.

Besides the CD40/CD40L pathway, the OX40/OX40L pathway has been investestigated as a possible therapeutic target in the treatment of RA. OX40 is known to be mainly expressed on activated T cells (including T follicular helper cells), as well as natural killer cells [108,109]. The interaction of OX40 and its ligand OX40L is known to be vital for Tfh cells to carry out their helper functions within GCs [110]. In the literature, both human and murine data suggest that the OX40/OX40L has a major role in the pathogenesis of RA [109,111,112,113]. With evidence from these studies, targeting the OX40/OX40L pathway might be beneficial for the treatment of RA. Thus far, data from murine models look promising. In CIA as well as autoimmune arthritis mice models, OX40L blockade was sufficient to significantly reduce bone and cartilage destruction as well as disease development [114,115]. In another arthritis mouse model, blocking the OX40/0X40 axis prevented the development of arthritis as well as a reduction in the number of Tfh cells in these mice [109]. Eventhough murine studies show a beneficial effect of blocking the OX40/OX40L pathway, to the best of our knowledge, there is no literature that has investigated the benefits of blocking the OX40/OX40L pathway in humans.

Another costimulatory pathway that has been investigated recently is the ICOS-ICOSL pathway. ICOS is a co-stimulatory molecule which is part of the CD28 family and is expressed in GC-Tfh cells. Within GCs, the interaction of ICOS on Tfh cells with its ligand ICOSL on B cells is crucial for Tfh cells to carry out their B cell helper functions within GCs. Moreover, ICOS was shown to be vital in maintaining the Tfh cell phenotype outside GCs [57] Nevertheless, the importance of ICOS in T cell-dependent B cell activation was shown in mice where lack of co-stimulation resulted in the formation of smaller GCs as well as reduced Tfh cell numbers [116,117]. Similarly, individuals deficient in ICOS presented with severe immunodeficiency [118]. Therefore, blocking the ICOS/ICOSL pathway might be beneficial therapeutically in autoimmune diseases. For instance in SLE, a phase Ib randomized double-blind study involving a monoclonal antibody against ICOSL reported potential efficacy and safety, supporting evidence of beneficial outcomes in ICOS blockade [119]. Thus far, targeting the ICOS/ICOSL pathway in RA mouse models looks promising in controlling Tfh activity. An earlier study blocking the ICOS/ICOSL pathway in the CIA mouse model reported beneficial effects in synovial tissues [120], further confirmed by Ronan O’Dwyer and colleagues who observed the therapeutic potential of ICOS blockade in CIA mice with an associated decrease in inflammation [121]. Similarly, in a model of G6PI-induced arthritis in DBA/1 mice, a transient blockade of the ICOS/ICOSL pathway was sufficient in reducing the severity of the disease as well as reducing levels of pro-inflammatory cytokines [122].

Taken together, blockade of the ICOS seems to be a promising direction to be exploited in RA treatment. However, current knowledge on the use of ICOS blockade in human RA seems to be lacking. Therefore, further investigation of ICOS blockade in humans is needed.

## 5. Questions Raised and Concluding Remarks

Although the literature reviewed herein provides compelling evidence implicating GCs in the generation of autoantibodies in RA, it is worth pointing out that several hypotheses, including a mucosal as well as a gut origin of autoantibodies in RA, have been in the spotlight for several years as well [123,124,125]. This raises an intriguing possibility that GCs located in lymphoid structures which can be found in these mucosal sites are the producers of these autoantibodies after all [126]. Therefore, recent high-throughput technologies coupled with advances in tissue sampling techniques will be crucial in unraveling the exact anatomic origin of autoantibodies in RA. Another question that needs an answer is whether aberrant Tfh cell signals alone are sufficient to dysregulate GCs, or whether the required dysregulation of GCs results from a combination with other underlying molecular events such as non-functional tingible body macrophages (TBMs) as well as defective GC checkpoints. 

A special interest might be located in the role of GCs in the pre-clinical phase of RA, which is characterized by the presence of ACPAs and/or RF in the absence of arthritis [127]. It is of interest that studies indicate that in this pre-clinical phase, diversification of the antibody response intensifies immediately before the onset of arthritis but also stops at onset [5]. Do GCs and various GC cellular subsets offer a biomarker potential for predicting the onset of RA in at risk individuals? Moreover, does insight into this process lead us to new targets for intervention? 

Therefore, and without doubts, GCs offer both the good, the bad and the ugly to human health. More insight into its role in health and autoimmunity may give us the tools to monitor, steer and rebalance the autoimmune state.

## Figures and Tables

**Table 1:** Summary of studies involving aberrant Tfh and Tfr cells in human RA.

Tfh Cells in RA	Findings
Arroyo-Villa et al. [65]	RA patients with active disease have a higher frequency of Tfh cells and a higher Tfh/Tfr ratio resulting from lower Tfr frequencies
Zhang et al. [66]	Increased frequencies of Tfh cells and IL-21 in RA patients which correlates positively with DAS28
Wang et al. [67], Zhou et al. [69]	Increased frequencies of Tfh cells in newly diagnosed RA patients correlating with activated B cells and DAS28
Niu et al. [68]	Elevated frequencies of Tfh cells, IL-21 and PD-1 in RA patients with active disease
Nakayamada et al. [70]	Higher proportions of Tfh cells in RA patients with active disease and, treatment with Abatacept reduces Tfh cell levels.
Su et al. [71]	Elevated frequencies of Tfh cells in RA patients compared to HCs
**Tfr cells in RA**	
Su et al. [71]	Decreased frequencies of Tfr cells in RA patients compared to HCs
Wang et al. [72]	Increased levels of Tfr cells in RA patients compared to HCs
Macdonald et al. [73]	Elevated percentages of Tfr cells in RA patients
Liu et al. [74]	Higher frequencies of Tfr and Tfr/Tfh cell ratio in RA patients with stable remission

RA; Rheumatoid Arthritis, Tfh; T follicular helper cells, Tfr; T follicular regulatory cells, PD-1; Program cell death 1, DAS28; Disease activity score evaluated in 28 joints, HCs; healthy controls.

## Data Availability

Not applicable.
